# A protocol for the development of PhyCaRe: An extension of the CARE guideline for physiotherapy using the Delphi method

**DOI:** 10.12688/f1000research.138599.2

**Published:** 2024-03-15

**Authors:** Waqar M. Naqvi, Gaurav Mishra, Aishwarya A. Pashine, Sakshi P. Arora, Sonia Gupta, Chanan Goyal, Ashish R. Varma, Zahiruddin Quazi, Ramprasad Muthukrishnan, Praveen Kumar Kandakurti, Laxmikant Umate

**Affiliations:** 1 Physiotherapy, Gulf Medical University, Ajman, United Arab Emirates; 2 Datta Meghe Institute of Higher Education and Research, Wardha, India; 3 Career College, Bhopal, India; 4 VSPM College of Physiotherapy, Nagpur, India; 5 Government Physiotherapy College, Raipur, India

**Keywords:** Case Report, CARE Guidelines, PhyCaRe, Physiotherapy, Physical Therapy, Reporting Guideline, Clinical Practice Guideline, CARE Extension

## Abstract

**Background:**

Case reports are one of the important forms of documentation and publication of clinical physiotherapy presenting the first line of evidence in scientific literature. In order to provide a systematic and precise structure for reporting and presenting cases, the CARE guidelines were established in 2013. However, these guidelines present limitations as while reporting require items of specific specialties following the checklist. Authors from different specialities have developed CARE extensions specifying the characteristic features of corresponding fields, however, an extension dealing with physiotherapy assessment and line of management in the CARE guidelines is proposed as PhyCaRe.

**Method:**

After consulting with the advisors, a draft will be prepared of the specific elements that should be included in the PhyCaRe using web Delphi methodology considering CARE statement as the source and SurveyMonkey will be used to undertake the web Delphi questionnaire. The web Delphi methodology will be assumed for three rounds and will be open to physiotherapists and others with substantial experience in reviewing case reports. Subsequently, an online consensus meeting, pilot testing, and submission of the CARE extension for physiotherapy will be conducted for publication.

**Dissemination:**

The 2010 “Guidance for Developers of Health Research Reporting” and instructions from the EQUATOR Network will be followed in the preparation of PhyCaRe guidelines. The guidelines will be propagated at different platforms and journals will be requested to adopt the guidelines.

**Registration:**

The reporting guideline under development is prospectively registered on the EQUATOR Network website on PhyCaRe – Reporting guideline for physiotherapy case reports.

**Abbreviations**
CINAHL: Cumulative Index to Nursing and Allied Health Literature
CR: Case Reports
FITT: Frequency, Intensity, Time, and Type
PT: Physiotherapy/Physical Therapy

## Introduction

The basic publishable unit in the healthcare sector is a case report (CR) which provides a detailed clinical course description of an individual patient. ^1^^–^^3^ A CR presents unusual signs and symptoms in rare diseases and syndromes with a novel and innovative approach of assessment and management. This avails the wide range of favourable or unfavourable outcomes of various approaches. ^4^ The trend of publishing CRs can be traced back to the early twentieth century and has been increasing ever since. ^2^^,^^5^

Physiotherapy or physical therapy (PT), is an integral part of healthcare sciences that deals with rehabilitation programs to relieve pain and restore functional independence, thus maximising the ability to participate. ^6^ The primary aim of PT is to treat and prevent physical impairments associated with injuries and optimise the capacity of an individual. ^7^ Physiotherapists evaluate, plan, and implement various rehabilitation programs by using a broad range of therapeutic techniques. Hence, they play a key role in restoring functional capacity of the patients and upgrading the quality of life. ^8^

Physiotherapists follow a thorough assessment of structural and functional impairments of different conditions with a note on positive findings for tailoring patient-oriented treatment goals. ^9^ However, from the assessment to the follow-up, the physiotherapeutic approach varies widely on the basis of confounding factors, the method of evaluation, frequency, intensity, time, and type (FITT) of the appropriate treatment as per individual needs. ^10^ This emphasises the significance of CR in the research literature creating an awareness about the profession in the scientific community.

In order to provide a framework pertaining to the uniformity and accuracy in the CR presentation, CARE guidelines were developed in 2013 ^11^ which consists of a 13-item checklist, thus satisfying the requirements of transparency as well as completeness in CRs. ^12^ Authors are obliged to follow CARE guidelines or at least a part of it applicable for a case while writing CRs. ^13^ There are over 10,000 published CRs in the PubMed database and more than 12,000 published CRs in the Scopus database, in accordance with the CARE guidelines and their present extensions. ^14^ However, the contribution of physiotherapy CRs is comparatively limited as compared to other healthcare fields. It may be because of the presenting lacuna in the checklist which is unable to cover the different aspects of physiotherapeutic evaluation, treatment, and follow-up. Moreover, one of the major limitations of CARE guidelines was that the CARE guidelines may possibly require extensions to include information particular to different specialties, practitioners, and patients. As a result, multiple extensions were developed for surgery ^5^; radiology ^13^; endodontic ^15^; therapeutic massage and bodywork ^16^; and acupuncture. ^17^ However, there is no such guideline specifying the checklist as per the needs of physiotherapeutic care.

Physiotherapists use different approaches for assessment as well as treatment like exercises, ^18^ electrotherapy, ^19^ manual therapy, ^20^ hydrotherapy, ^21^ and gamification ^22^ wherein the effective dosage varies with patients to the manifesting conditions extensively necessitating the use of an associated format for effective documentation and presentation, so that the claims will benefit the researchers, academicians, and clinicians around the globe. ^3^ Furthermore, this will provide an uniform, structured, and systematic way of presenting physiotherapeutic approach and efficacy in terms of FITT’s principle. ^10^ This will enhance the reproducibility of assessment and management in clinical and/or community settings. This protocol aims at developing an extension dealing with PT assessment and line of management in the CARE guidelines as PhyCaRe by adding new items and adapting the current ones, adding to the EQUATOR Network’s guidelines while considering the 2010 “Guidance for Developers of Health Research Reporting”.

## Methods

### Initiation of the project

**Executive group**

The executive group consisting of co-authors will supervise the conduction of the required assessment, drafting of primary items; arranging, and executing the web Delphi method; concluding and finalising the draft; coordinating the preparations of the final manuscript and CARE extension for physiotherapy as PhyCaRe. The members of the web Delphi panel will include physiotherapists, clinical practitioners, journal editors, peer reviewers, academic editors, researchers, statisticians, methodologists, epidemiologists, content experts, and professional medical writers who will be invited to attend at least three rounds of the web Delphi process. ^23^

**Advisory group**

This team will help with methodology enquiries, aid the executive team in recognizing key web Delphi panel members, offer suggestions as required, and include experts in the field of PT, guideline development, and CR publishing. The patient representatives to the group will also be invited if possible. The key members of EQUATOR guidelines who have participated in reporting guideline development groups at least once in the past year will also be invited. Next, each member who accepted our invitation will be requested to suggest additional potential members (beyond those identified in our list).

**Registration**

The reporting guideline under development is registered on the EQUATOR Network website as PhyCaRe – Reporting guideline for physiotherapy case reports.

### Pre-consensus

**Literature review**

For reviewing the existing literature, published PT CRs will be searched and extracted. After evaluating and assessing these CRs, the primary items for the web Delphi survey method will be drafted.

**Identify physiotherapy reporting standards**

The data will be searched and extracted from six different databases: PEDro, PubMed-PubMed Central-MEDLINE, Scopus, Embase, Cumulative Index to Nursing and Allied Health Literature (CINAHL), and Google Scholar. Additional searches on the EQUATOR network website, as well as tracking of the citations and references of the acquired studies will be performed. Any previous version of the reporting guidelines will be excluded and PT reporting guidelines will be included in the process. A free and combination of keywords will be used to search for the required data. In order to conduct the process, the PRISMA-S guideline will be assigned and the search items will include, “CARE guideline, reporting, case report, case study, case presentation, physiotherapy, physical therapy, rehabilitation, exercises, electrotherapy, manual therapy, therapeutic exercise, exergaming, gamification, functional independence, quality of life, health intervention”.

**Finding recent physiotherapy case reports**

The literature will be searched in the above mentioned databases with the use of selected keywords. CRs literature will be included whereas, non-clinical researches will be excluded from the study.

**Drafting of primary items**

The executive group will search for the results and draft the primary elements specific to PT prior to the confirmation from the advisory committee. These primary specific elements will then be evaluated by the web Delphi panel members.

### Delphi consensus methodology

A survey will be conducted using standard web Delphi methodology for which SurveyMonkey ^®^ ( www.surveymonkey.com) will be employed to administer the web Delphi questionnaire. ^5^^,^^1^^,^^3^ All the stakeholders will complete the same questionnaire throughout the process. The web Delphi questionnaire is supposed to comprise two sections. The respondents will be asked to rank the “importance” and “appropriateness” of each element in the first section. In the second section, the respondents will be asked to suggest new items or adaptations of the existing items in the CARE statement to fulfil the specific requirements of CRs in PT. Accordingly, the above mentioned suggestions will be included in the first round which will be oriented towards generating new items and adapting the current ones for PT.

A nine-point Likert scale will be employed in each consecutive round wherein respondents will rank the relevance of each outcome’s reporting. ^5^^,^^1^^,^^3^ The outcome of limited relevance is signified by 1 to 3; 4 to 6 signifies that the outcome is relevant but not essential; and 7 to 9 denotes a relevant and essential outcome. Only when at least 70% of respondents will rate an item 7 to 9 and less than 15% will rate it 1 to 3, the item will be promoted in the reporting standards. Similarly, if 70% or more of the respondents scored an item from 1 to 3 and 15% or less scored it from 7 to 9, such an outcome will be rejected from the reporting guidelines. Items with scores of 4 to 6 will be revised based upon the comments of the advisory group members for the next round of the web Delphi methodology. Administration of the questionnaire as well as the sequential rounds will continue unless a comprehensive set of findings with concurred definitions is obtained. Due to the fact that there is no requirement set for number of web Delphi rounds, the entire procedure will be carried out online. However, according to our expectations, at least three web Delphi rounds will be required. Furthermore, the same procedure will be employed to get accepted definitions for the results. ^23^

### Statistical analysis

Following the conclusion of each round, analysis will be conducted to determine if each item will be included, excluded, or forwarded for the next round. To determine which category suggested elements will be added, thematic analysis of the reviews will be done and descriptive statistics (median and interquartile range) will be used to analyse the Likert items.

### Generating list of elements for consideration at the online consensus meeting

Following each round of the web Delphi procedure, the executive group will collect the completed questionnaires, compile the results, and summarise the respondents’ comments and suggestions regarding any adaptations to the CARE guidelines and new items for the PhyCaRe. If no consensus will be reached, the web Delphi exercise will be continued for another round. Once consensus will be reached, the executive group will prepare a list of items for consideration by the advisory group at the meeting.

### Prepare for the online consensus meeting

**Venue and duration**

Face-to-face online consensus meetings will be held through a teleconference platform. The meeting participants will be anticipated to reach a consensus on the preliminary version of the PhyCaRe checklist in about one day. Therefore, a one-day online consensus meeting will be hold.

**Agenda**

The executive group will develop an agenda for online meetings and circulate it in advance to all participants before the meeting. The overall framework of the agenda will include the PhyCaRe statement background, a preliminary version of the PhyCaRe reporting checklist, the process of the online consensus meeting, and a discussion on the possibility of developing a flow diagram for the PhyCaRe statement.

**Online consensus meeting**

The executive group will create a checklist of the included items after the web Delphi process and will host an online consensus conference to review the outcomes. The meeting will be attended by the members of the web Delphi panel who will be participating in each round of the web Delphi procedure.

In order to set the stage for the discussion, first a brief outline of the background, methodology, and outcomes of the web Delphi process will be provided. Second, a consultation to update each item on the checklist will be presented. Third, voting will be conducted on each suggested item and phrasing. The experts will review the checklist of items once more after the meeting to ensure that their inputs are properly received and taken into account. The CARE guideline and the EQUATOR template will then be used to generate the draft of the guideline.

### Post-consensus

**Drafting and finalising the checklist and developing guidance statement**

The PhyCaRe executive group will begin to draft the final checklist immediately after the virtual meeting. The executive group will work in parallel on the PhyCaRe statement. In the PhyCaRe statement, an explanation will be provided on which parts of the PhyCaRe checklist have been taken unchanged from the original CARE checklist and which items have been modified or added in response to the specific requirements of PT.

**Consulting advisors**

To further discuss, refine the terminology, and organisation of the checklist, the manuscript will be delivered to the advisory team for reviewing, and after receiving their feedback the amended version of the PhyCaRe will be employed for the pilot test.

**Pilot test of the checklist**

A pilot test of the PhyCaRe checklist among potential users will be conducted for validation which include physiotherapists, clinicians, and editors of journals publishing CRs on PT, and intend to collect their feedback on the checklist. Using the PhyCaRe checklist, the reporting precision of a sample of CR will be evaluated which is published in 2022 with an intention to search for any specific issues with any of the presenting or missing elements. Additionally, an online survey of the CR’s corresponding authors for assessing the ease of execution with employment of PhyCaRe will also be conducted. The executive group will review the comments and incorporate them as appropriate into future revisions of the PhyCaRe checklist. A thorough critical appraisal will be undertaken to map the final draft of the guidelines after receiving feedback from piloting.

**Elaboration of explanatory document**

To enable accurate application of PhyCaRe, a comprehensive descriptive and explanatory material will be provided.

**Development of publication strategy**

The executive group will facilitate the journal submission and will work with the advisory group to discuss suitable journals, prepare a manuscript that all authors agree on, and submit the final manuscript to a set of journals for simultaneous publication. The executive group will communicate with the editors of corresponding journals before submission to facilitate multiple publications and will publish a comprehensive protocol, checklist, and background information on PhyCaRe in the form of an academic paper.

### Post-publication activities

**Receiving the feedback and amending as appropriate**

Feedback on PhyCaRe will be received from different individuals, including those involved in establishing the guidelines, peer-reviewed journals that publish PT cases using PhyCaRe, clinical PT practitioners, researchers, etc.

**Encouraging guidelines endorsement**

The editors of PT journals will be involved in the development of the PhyCaRe checklist to facilitate its endorsement by journals. PT journals that publish CRs will be recommended to include the PhyCaRe checklist in their guidance for authors, and fulfill the expectations regarding the requirements of PT CRs. Additionally, the reporting checklist on the EQUATOR website or library will be disseminated and recommended to the researchers to report CRs on PT through academic conferences or operational groups using PhyCaRe.

**Supporting adherence to the guidelines**

The critiques and feedbacks will be continuously collected and reviewed about the precision and accuracy of PhyCaRe checklist. The suggestions will be used to maximise adherence to the purpose of the checklist by incorporating critical feedback and productive analysis into revisions of the updated checklist.

**Monitoring and evaluating the impact of reporting guidelines**

The observation and evaluation of the impact of reporting guidelines will be conducted by following a three-step process. The first phase will detect how journals of the CR for PT will be accepting and listing in the journal policy. The second phase will involve estimation of the overall number of PT CRs that adhere to the PhyCaRe following a qualitative analysis of the CR. Third, CRs adhering to the PhyCaRe guidelines and CRs not adhering to the criteria will be compared. Further, a questionnaire-based survey will be carried out with stakeholders to find out their awareness and use of PhyCaRe.

**Updating the guidelines**

On the basis of stakeholders feedback and the findings of the monitoring and assessment of the PT statement after five years, a meeting or assembly of the relevant experts will be held with the goal of updating PhyCare.

## Discussion

The purpose of this protocol is to develop a PT specific CARE guideline extension as PhyCaRe, by conducting a web Delphi survey method in which collective opinions of a group of experts will be gathered to identify the reporting items and to develop reporting guidelines for CRs in PT. ^5^^,^^1^^,^^3^ CARE guideline extensions have been developed and validated for various specialties and super specialities of healthcare sciences. Agha *et al.* developed the Surgical CAse REport (SCARE) guidelines using a consensus based approach with an aim of adding the surgical perspective while reporting a CR. ^5^ Similarly, Nagendrababu et al. developed the Preferred Reporting Items for Case Reports in Endodontics (PRICE) guidelines as an extension of the CARE guidelines for specifying the endodontics application. ^15^ Van Haselen developed the HOM-CASE guideline extension which substantially improved the quality of reporting homoeopathic clinical cases along with the consideration and follow up during the treatment. ^24^ In addition, Munk *et al.* formed a Therapeutic Massage and Bodywork (TMB) CR template which has documented and presented the impact of the TMB. ^16^ Furthermore, Duan *et al.* has also proposed a protocol for development of guidelines for accurate reporting of clinical cases of acupuncture. ^17^

Reporting guidelines for specific areas could enhance the quality of the corresponding research and promote its dissemination and transparency but will only do so if the methods used to develop them are scientific, disciplined, and transparent. ^11^ This protocol reports the methodologies that will be employed to develop PhyCaRe guidelines. The supporting systematic review is under process to recognize the lacuna in the existing reporting criterias. However, the methodologies, protocols, and content of the development of other extended versions of the CARE guidelines are reviewed and critically appraised for differentiating the required items in the PhyCaRe checklist. A face-to-face online consensus meetings will be hold for there is no set number of web Delphi rounds, but it can be easily conuducted with an active involvement of the panel without offline meetings. A detailed description of the expert group is yet to be disclosed as it has not yet been assembled. However, these limitations will be considered as not to affect the quality of the PhyCaRe guidelines.

CRs drafted in accordance with PhyCaRe will provide valuable evidence from the point of care, thus enhancing the accuracy and transparency of PT care for clinicians, patients, policy makers, researchers, and medical journals. Busy professionals would be encouraged to publish more CRs to share their unique experiences and encounters as PhyCaRe will save time in drafting manuscripts for publication targeting reputed journals.

## Data Availability

No data associated with this article.
