# A protocol for the development of PhyCaRe: An extension of the CARE guideline for physiotherapy using the Delphi method

**DOI:** 10.12688/f1000research.138599.3

**Published:** 2025-04-14

**Authors:** Waqar M. Naqvi, Gaurav Mishra, Aishwarya A. Pashine, Sakshi P. Arora, Sonia Gupta, Chanan Goyal, Ashish R. Varma, Zahiruddin Quazi, Ramprasad Muthukrishnan, Praveen Kumar Kandakurti, Laxmikant Umate

**Affiliations:** 1 Physiotherapy, Gulf Medical University, Ajman, United Arab Emirates; 2 Datta Meghe Institute of Higher Education and Research, Wardha, India; 3 Career College, Bhopal, India; 4 VSPM College of Physiotherapy, Nagpur, India; 5 Government Physiotherapy College, Raipur, India

**Keywords:** Case Report, CARE Guidelines, PhyCaRe, Physiotherapy, Physical Therapy, Reporting Guideline, Clinical Practice Guideline, CARE Extension

## Abstract

**Background:**

Background Case reports are one of the important forms of documentation and publication of clinical physiotherapy presenting the first line of evidence in scientific literature. In order to provide a systematic and precise structure for reporting and presenting cases, the CAse REport (CARE) guidelines were established in 2013. However, these guidelines present limitations as reporting requires items of specific specialties following the checklist. Authors from different specialities have developed CARE extensions specifying the characteristic features of corresponding fields, however, an extension dealing with physiotherapy assessment and line of management in the CARE guidelines is proposed as Physiotherapy CAse REport (PhyCARE).

**Method:**

A survey will be conducted using standard e-Delphi methodology using Google forms®. ^1^^,^^3^^,^^5^ The e-Delphi questionnaire shall comprise responses on the relevance of each item of the CARE 2013 guideline with respect to physiotherapy. This shall be followed by suggestions from e-Delphi members regarding the incorporation of new items or adaptations of the existing items in the CARE 2013 statement using controlled feedback. Accordingly, based on the responses and suggestions from the e-Delphi experts, the core committee will draft the initial version of the PhyCARE checklist, which will be included in the second round of the e-Delphi process. Administration of the questionnaire as well as the sequential rounds shall continue unless a comprehensive set of findings with concurred definitions are obtained. Due to the fact that there is no requirement set for the number of e-Delphi rounds, the entire procedure will be carried out online. Subsequently, followed by pilot testing, submission of the CARE extension for physiotherapy (PhyCARE) will be conducted for publication and dissemination. The 2010 “Guidance for Developers of Health Research Reporting” and instructions from the Enhancing the QUAlity and Transparency Of health Research (EQUATOR) Network will be followed in the preparation of PhyCARE guidelines. The guidelines will be propagated on different platforms and journals will be requested to adopt the guidelines.

**Registration:**

The reporting guideline under development is prospectively registered on the EQUATOR Network website on PhyCaRe – Reporting guideline for physiotherapy case reports.

**Abbreviations**
CINAHL: Cumulative Index to Nursing and Allied Health Literature
CR: Case Reports
FITT-VP: Frequency, Intensity, Time, Type, Volume, and Progression
PT: Physiotherapy/Physical Therapy

## Introduction

The basic publishable unit in the healthcare sector is a case report (CR) which provides a detailed clinical course description of an individual patient. ^1^^–^^3^ A CR presents unusual signs and symptoms in rare diseases and syndromes with a novel and innovative approach to assessment and management. This avails the wide range of favourable or unfavourable outcomes of various approaches. ^4^ The trend of publishing CRs can be traced back to the early twentieth century and has been increasing ever since. ^2^^,^^5^

Physiotherapy or physical therapy (PT), is an integral part of healthcare sciences that deals with rehabilitation programs to relieve pain and restore functional independence, thus maximising the ability to participate. ^6^ The primary aim of PT is to treat and prevent physical impairments associated with injuries and optimise the capacity of an individual. ^7^ Physiotherapists evaluate, plan, and implement various rehabilitation programs by using a broad range of therapeutic techniques. Hence, they play a key role in restoring the functional capacity of the patients and upgrading the quality of life. ^8^

Physiotherapists follow a thorough assessment of structural and functional impairments of different conditions with a note on positive findings for tailoring patient-oriented treatment goals. ^9^ However, from the assessment to the follow-up, the physiotherapeutic approach varies widely on the basis of confounding factors, the method of evaluation, frequency, intensity, time, type, volume, and progression (FITT-VP) of the appropriate treatment as per individual needs. ^10^ This emphasises the significance of CR in the research literature creating an awareness about the profession in the scientific community.

In order to provide a framework pertaining to the uniformity and accuracy in the CR presentation, CAse REport (CARE) guidelines were developed in 2013 ^11^ which consists of a 13-item checklist, thus satisfying the requirements of transparency as well as completeness in CRs. ^12^ Authors are obliged to follow CARE guidelines or at least a part of it applicable for a case while writing CRs. ^13^ There are over 10,000 published CRs in the PubMed database and more than 12,000 published CRs in the Scopus database, in accordance with the CARE guidelines and their present extensions. ^14^ However, the contribution of physiotherapy CRs is comparatively limited as compared to other healthcare fields. It may be because of the presenting lacuna in the checklist which is unable to cover the different aspects of physiotherapeutic evaluation, treatment, and follow-up. Moreover, one of the major limitations of CARE guidelines was that the CARE guidelines may possibly require extensions to include information particular to different specialties, practitioners, and patients. As a result, multiple extensions were developed for surgery ^5^; radiology ^13^; endodontic ^15^; therapeutic massage and bodywork ^16^; and acupuncture. ^17^ However, there is no such guideline specifying the checklist as per the needs of physiotherapeutic care.

Physiotherapists use different approaches for assessment as well as treatment like exercises, ^18^ electrotherapy, ^19^ manual therapy, ^20^ hydrotherapy, ^21^ and gamification ^22^ wherein the effective dosage varies with patients to the manifesting conditions extensively necessitating the use of an associated format for effective documentation and presentation so that the claims will benefit the researchers, academicians, and clinicians around the globe. ^3^ Furthermore, this will provide a uniform, structured, and systematic way of presenting physiotherapeutic approach and efficacy in terms of frequency, intensity, time, type, volume, and progression (FITT-VP) principle. ^10^ This will enhance the reproducibility of assessment and management in clinical and/or community settings. This protocol aims at developing an extension of the CARE guideline as Physiotherapy CAse REport (PhyCARE) dealing with PT assessment and line of management by adding new items and adapting the current ones, contributing to the Enhancing the QUAlity and Transparency Of health Research (EQUATOR) Network’s guidelines while considering the 2010 “Guidance for Developers of Health Research Reporting”.

## Methods

### Registration

The reporting guideline under development PhyCARE is registered with the EQUATOR network ^23^ and ethical approval for the PhyCARE project was obtained from the Datta Meghe Institute of Higher Education and Research, India (DMIHER (DU)/IEC/2023/1051).

### Pre-consensus

#### Literature review

For reviewing the existing literature, published PT CRs will be searched and extracted to assess the common gaps or inconsistencies reported.

### Identify physiotherapy reporting standards

Searches on the EQUATOR network website, as well as tracking of the citations and references of the acquired studies, will be performed. Any previous version of the reporting guidelines will be excluded and PT-specific reporting guidelines will be included in the process. A free combination of keywords will be used to search for the required data. The search items will include, “CARE guideline”, “reporting”, “case report”, “case study”, “case presentation”, “physiotherapy”, “physical therapy”, “rehabilitation”, “exercises”, “electrotherapy”, “manual therapy”, “therapeutic exercise”, “exergaming”, “gamification”, “functional independence”, “quality of life”, “health intervention”.

### Identifying recent physiotherapy case reports and assessing their quality of reporting

The search will be limited to PT CRs published within the specified time frame (e.g., last five years). Non-clinical research or articles not specified as CRs will be excluded. After identification of the specific CRs, their quality will be assessed to determine frequently missing data with respect to PT-specific elements.

### Identification of panel experts

#### Core committee

The core group consisting of co-authors shall supervise the conduction of the required assessment, drafting of primary items; arranging, and executing the e-Delphi method; concluding and finalising the draft; coordinating the preparations of the final manuscript and CARE extension for physiotherapy as PhyCARE. The members of the e-Delphi panel shall include physiotherapists, clinical practitioners, journal editors, peer reviewers, academic editors, researchers, statisticians, methodologists, epidemiologists, who shall be invited to participate in e-Delphi rounds. ^24^ Each member who has formally accepted the invitation will be requested to suggest additional potential members (beyond those identified in our list).

### E-Delphi consensus methodology

A survey will be conducted using standard e-Delphi methodology using Google forms®. ^1^ ^,^ ^3^ ^,^ ^5^ The e-Delphi questionnaire shall comprise responses on the relevance of each item of the CARE 2013 guideline with respect to PT by using two options *‘Relevant’* or *‘Not Relevant’,* whilst the suitability of each item will be evaluated using a 5-point Likert scale (1 = strongly disagree to 5 = strongly agree). ^25^ This shall be followed by suggestions from e-Delphi members regarding the incorporation of new items or adaptations of the existing items in the CARE 2013 statement using controlled feedback. Accordingly, based on the responses and suggestions from the e-Delphi experts, the core committee will draft the initial version of the PhyCARE checklist, which will be included in the second round of the e-Delphi process. This round will be oriented towards generating new items and adapting the current ones followed by consecutive rounds if required.

A five-point Likert scale shall be employed in each consecutive round wherein respondents will rank the relevance of each outcome’s reporting. ^1^ ^,^ ^3^ ^,^ ^5^ ^,^ ^25^An item will be included automatically if more than 70% of respondents rate it atleast between 4 to 5. Moreover, items with moderate support that do not reach the above threshold (e.g., 50-69% rating 3-5) will be revised based on qualitative comments and shall be reintroduced in the next round. However, items that fail to meet the consensus criteria (e.g., less than 49% of respondents rating 1 to 2 will be excluded, unless strong expert feedback indicates significant rationale for another revision. Administration of the questionnaire as well as the sequential rounds shall continue unless a comprehensive set of findings with concurred definitions are obtained. Due to the fact that there is no requirement set for the number of e-Delphi rounds, the entire procedure will be carried out online. ^24^ ^,^ ^25^

### Statistical analysis

Following the conclusion of each round, analysis will be conducted to determine if each item should be included, excluded, or revised for the next round and controlled feedback will be provided to the panel experts. A thematic analysis of the open-ended comments or suggestions shall be conducted and descriptive statistics (median and interquartile range) will be used to analyse the Likert scores of each item.

### Generating a list of elements for consideration

Following each round of the e-Delphi procedure, the core committee shall amalgamate the completed questionnaires, compile results (quantitative ratings and thematic analysis), and summarise respondents’ feedback regarding any adaptations to the CARE 2013 guidelines and incorporation of new items for the PhyCARE. If no consensus is achieved, the e-Delphi exercise will be continued for another round. Once consensus is achieved, the core committee shall prepare the final draft of accepted items constituting the PhyCARE guideline.

### Post-consensus

#### Drafting and finalising the checklist and developing guidance statement

The PhyCARE core committee will begin to draft the final checklist immediately after the concluding round (consensus achieved) of the e-Delphi process. The core group will work in parallel on the PhyCARE statement. In the PhyCARE statement, an explanation will be provided on which parts of the PhyCARE checklist have been taken unchanged from the original CARE checklist and which items have been modified or added in response to the specific requirements of PT.

#### Pilot test of the checklist

A pilot test of the PhyCARE checklist among potential users will be conducted for validation which includes physiotherapists, clinicians, and editors of journals publishing CRs on PT and to collect their feedback on the checklist. Using the PhyCARE checklist, the reporting precision of a sample of CRs will be evaluated which is published with an intention to search for any specific issues with any of the presenting or missing elements. An online survey executed by the core committee will be performed to collect their experiences, feedback, or suggestions. Based on the feedback received, the core committee will review the comments and incorporate them as appropriate to revise the PhyCARE guideline as required. A thorough critical appraisal will be undertaken to map the final draft of the PhyCARE guideline after receiving feedback from piloting.

#### Elaboration of explanatory document

To enable accurate application of PhyCARE, comprehensive descriptive and explanatory document material will be provided.

#### Development of publication strategy

The core committee will facilitate the journal submission after discussion on suitable journals, prepare a manuscript that all authors agree on, and submit the final manuscript to a set of journals for simultaneous publication. The core committee will communicate with the editors of corresponding journals before submission to facilitate multiple publications and will publish a comprehensive protocol, checklist, and background information on PhyCARE in the form of an academic paper.

### Post-publication activities

#### Receiving the feedback and amending as appropriate

Feedback on PhyCARE will be received from different individuals, including those involved in establishing the guidelines, peer-reviewed journals that publish PT cases using PhyCARE, clinical PT practitioners, researchers, and other healthcare professionals.

#### Encouraging guidelines endorsement

To facilitate PhyCARE endorsement by journals that publish CRs, the editors will be recommended to include the PhyCARE checklist in their “Instructions for authors” section and fulfill the expectations regarding the requirements of PT CRs. Additionally, the reporting checklist on the EQUATOR website or library will be disseminated and will be recommended to the researchers to report CRs on PT using PhyCARE through academic conferences or operational groups.

#### Supporting adherence to the guidelines

The critiques and feedback will be continuously collected and reviewed about the precision and accuracy of the PhyCARE checklist. The suggestions will be used to maximise adherence to the purpose of the checklist by incorporating critical feedback and productive analysis into revisions of the updated PhyCARE checklist.

#### Monitoring and evaluating the impact of reporting guidelines

The observation and evaluation of the impact of reporting guidelines will be conducted by following a three-step process. The first phase will detect how journals publishing CRs for PT accept and list the PhyCARE checklist in the journal policy. The second phase will involve the estimation of the overall number of PT CRs that adhere to the PhyCARE following a qualitative analysis of the CRs. Third, CRs adhering to the PhyCARE guidelines and CRs not adhering to the criteria will be compared. Further, a questionnaire-based survey will be carried out with stakeholders to find out their awareness and use of PhyCARE.

#### Updating the guidelines

On the basis of stakeholder's feedback and the findings of the monitoring and assessment of the PhyCARE statement after five years, a meeting or assembly of the relevant experts will be held with the goal of updating PhyCARE.

## Discussion

The purpose of this protocol is to develop a PT specific CARE guideline extension as PhyCARE, by conducting an e-Delphi survey method in which collective opinions of a group of experts will be gathered to identify the reporting items and to develop reporting guideline for CRs in PT. ^1^ ^,^ ^3^ ^,^ ^5^ CARE guideline extensions have been developed and validated for various specialities and super specialities of healthcare sciences. Agha *et al.* developed the Surgical CAse REport (SCARE) guidelines using a consensus based approach to add the surgical perspective while reporting a CR. ^5^ Similarly, Nagendrababu *et al*. developed the Preferred Reporting Items for Case Reports in Endodontics (PRICE) guidelines as an extension of the CARE guidelines for specifying the endodontics application. ^15^ Van Haselen developed the HOM-CASE guideline extension which substantially improved the quality of reporting homoeopathic clinical cases along with the consideration and follow up during the treatment. ^26^ In addition, Munk *et al.* formed a Therapeutic Massage and Bodywork (TMB) CR template which has documented and presented the impact of the TMB. ^16^ Moreover, Duan *et al.* have also proposed a protocol for the development of guidelines for accurate reporting of clinical cases of acupuncture. ^17^ Furthermore, Wang M *et al.* reported a radiology extension of the CARE guideline for radiological CRs. ^27^

Reporting guidelines for specific areas could enhance the quality of the corresponding research and promote its dissemination and transparency but will only do so if the methods used to develop them are scientific, disciplined, and transparent. ^11^ This protocol reports the methodologies that will be employed to develop PhyCARE guidelines. The supporting literature review is under process to recognize the lacuna in the existing reporting criterias. However, the methodologies, protocols, and content of the development of other extended versions of the CARE guidelines are reviewed and critically appraised for differentiating the required items in the PhyCARE checklist. A detailed description of the expert group is yet to be disclosed as it has not yet been assembled. However, these limitations will be considered as not to affect the quality of the PhyCARE guidelines.

CRs drafted in accordance with PhyCARE will provide valuable evidence from the point of care, thus enhancing the accuracy and transparency of PT care for clinicians, patients, policymakers, researchers, and medical journals. Busy professionals would be encouraged to publish more CRs to share their unique experiences and encounters as PhyCARE will save time in drafting manuscripts for publication targeting reputed journals.

## Data Availability

No data associated with this article.
