# Supplementary material for: A protocol for the development of PhyCaRe: An extension of the CARE guideline for physiotherapy using the Delphi method
Source: F1000Res. 2025 Apr 14;12:838. Originally published 2023 Jul 17. [Version 3] doi: 10.12688/f1000research.138599.3 (PMC10465994; doi:10.12688/f1000research.138599.3)
Supplement: Peer review history

# Reviewer report: Reviewer response for version 1

**DOI:** 10.5256/f1000research.151809.r197550

**Published:** 2023-08-29

**Authors:** Soheil Mansour Sohani

**Affiliations:** 1 Department of physical therapy, school of rehabilitation sciences, Iran university of medical sciences, Tehran, Iran

This study introduces the rationale, research questions, and the necessity behind this. The existing reporting guidelines for physiotherapy are outlined, along with their limitations. This proposal identified an overlooked opportunity to enhance the CARE checklist for physiotherapy, which has the potential to revolutionise the reporting of physiotherapy efficacy and patient care. Methodology section meticulously outlines the steps involved in the Delphi process. This covers the planning, approach, execution, and outcome analysis of the Delphi procedure. The discussion section delves into various aspects that underscore the need for this extension. Additionally, the proposal anticipates the future implications of this extension. Although I would like to suggest the authors if they can add the word “Web” to the Delphi process and it will be more suited for the methodology. There is one minor suggestion in the registration section of the manuscript. It would be better to replace “live” with registered words so that sentences are more scientifically sound. Upon critical assessment, the article is deemed scientifically suitable for publication.

Is the study design appropriate for the research question?

Yes

Is the rationale for, and objectives of, the study clearly described?

Yes

Are sufficient details of the methods provided to allow replication by others?

Yes

Are the datasets clearly presented in a useable and accessible format?

Not applicable

Reviewer Expertise:

NA

I confirm that I have read this submission and believe that I have an appropriate level of expertise to confirm that it is of an acceptable scientific standard.

# Response

**Published:** 2024-03-12

**Authors:** Dr. Waqar Naqvi

Dear Dr. Sohani,

 Greetings, 

 Thank you for your review. We have amended your suggestions in the protocol. 

 Regards,

 Waqar

# Reviewer report: Reviewer response for version 1

**DOI:** 10.5256/f1000research.151809.r188236

**Published:** 2023-07-27

**Authors:** Amna Khan

**Affiliations:** 1 Department of Physical Therapy, Ziauddin College of Rehabilitation Sciences, Ziauddin University, Karachi, Sindh, Pakistan

The rationale, research question, and the need of the study has been presented with a precise description of limitations in the existing reporting guidelines corresponding to the Physiotherapy. This proposal identified and addressed an extension to the CARE checklist for Physiotherapy which went unnoticed till now and the initiative would bring a revolutionary change in reporting the efficacy and patient care aspect of the physiotherapy.

 The methodology has described each and every step of undertaking of Delphi process concisely. The details have been provided with the planning, approach, conduction, and analysis of the outcome of Delphi procedure. The discussion has described the different aspects prompting to the need of this extension. The proposal is written with a perspective of determining the future implication of the extension too.

 For critically appraising the article, the article sounds suitable for indexing.

Is the study design appropriate for the research question?

Yes

Is the rationale for, and objectives of, the study clearly described?

Yes

Are sufficient details of the methods provided to allow replication by others?

Yes

Are the datasets clearly presented in a useable and accessible format?

Not applicable

Reviewer Expertise:

NA

I confirm that I have read this submission and believe that I have an appropriate level of expertise to confirm that it is of an acceptable scientific standard.

# Response

**Published:** 2024-03-12

**Authors:** Dr. Waqar Naqvi

Dear Dr. Khan,

 Greetings, 

 Thank you for your review. We have amended your suggestions in the protocol. 

 Regards,

 Waqar
